# Toward a Model of Work-Related Self: A Narrative Review

**DOI:** 10.3389/fpsyg.2016.00331

**Published:** 2016-03-08

**Authors:** Igor Knez

**Affiliations:** Social Work and Psychology, University of GävleGävle, Sweden

**Keywords:** work-related identity, work-related-cognition, work-related emotion, work-related satisfaction, work-related meaning, work-related self

## Abstract

Occupational work as personal and social identification can be conceptualized as one of the life goals that we strive for and find meaning in. A basic categorization of the phenomenon of work-related identity is suggested, based on psychological theories of identity, memory and relational schema. It distinguishes between organizational, workgroup and professional identity. The two former relate to the concepts of social identity and collective self and the latter to the concepts of personal identity and individual self. These are assumed to form functionally independent cognitive structures, leading to separate motivations and influences on work-related satisfaction. Given this, empirical research on the impact of work-related identity on employee satisfaction, in general terms, is reviewed. The article concludes with some prospective directions for future research by sketching a general model of work-related self. It is hypothesized to evolve by a causal progression from employment across time via emotional and cognitive components.

## Introduction

Employment is central to our lives. A large part of our waking life is related to occupational work and its life-long self-related construction (Guichard, 2009) and meaning (Cochran, 1991). It gives us social and economic status and brings prosperity to the common collective (Super, 1990). Organized labor also forms the foundation for personal health, development, and individual and social identity (Carr et al., 2003; Parker et al., 2003; Paul and Moser, 2009). According to Blustein (2006), it will gain even greater importance in the future due to employment’s even more changeable form and content.

Humans are the only species that can plan for the future and meta-cognitively reason and reflect upon goals (type of “possible selves” – Markus and Nurius, 1986; Vignoles et al., 2008) such as those related to present and future employment. Thus, we do not, as other types of animal, relate only to the present needs of food and shelter. We think about and plan for long-term goals which may give us the *meaning* of, basically meaningless, life; at least according to existential philosophy (Camus, 1942). One such life-long objective is work *per se*; as Butler (1998, p. 70) pointed out: “Every man’s [woman’s – added] work… is always a portrait of himself [herself – added].”

We have all been asked: What will you *be* when you grow up? When we meet old friends that we have not seen for a long time, or when we, for that matter, get to know new people, we are often faced with similar questions: What do you *do* nowadays? An answer to such a question indicates our individual and social affinities, as well as the socio-economic category of these ties (Fouad and Bynner, 2008). Thus, and generally speaking, occupational work as personal and social identification, the career, can be conceptualized as one of the life goals that we strive for and find meaning in (Gini, 1998), analogous with Sisyphus rolling the boulder up the hill (Camus, 1942).

How we implement ourselves into work roles across a lifetime have been addressed by, for example, the career accounts (e.g., Cochran, 1992, 1997; Savickas, 2001, 2002, 2005) suggesting that a career is a “superordinate construct that allows people to construct connections among actions; to account for effort, plans, goals, and consequences” (Young et al., 2002, p. 2017). This phenomenon is also referred to as a type of self-related narrative, a “storied self” or a “narrative identity” (McAdams, 1995, 2006). It comprises recollections of our work- and life-related experiences operating continuously in person-environment interplay (Cochran, 1997; Bruner, 2004; Savickas et al., 2009) of interpersonal relationships, work roles and personal careers (Flum, 2001; Blustein, 2001; Blustein et al., 2004). For that reason, the concept of identity and the processes of identity adjustments and reconstructions are central to the career theory; accordingly, suggesting that we form and share our careers through our self-constructing narratives (Bujold, 2004; Di Fabio and Maree, 2013; Di Fabio and Bernaud, 2014).

### Aims of Review

Corresponding, in general terms, to Ashforth et al. (2008) four fundamental identity-related questions: “What is identity?”; “Why does identity matters?”; How does identity evolve?”; and “Are there many or one identity?” this article has four aims. Firstly I review the research on identity, memory and the self (“What is identity and how does it evolve?”). I then review the research on work-related identity (“Are there many or one identity?”) and work-related identity in practice (“Why does identity matters?”).

Finally and given the previous reviews, I suggest a model of work-related self; “an individual work identity” (Walsh and Gordon, 2008). It includes an emotional component (involving process of work-related attachment/belonging/closeness) and a cognitive component (involving processes of work-related coherence, correspondence, temporality/mental time, reflection, agency). Work-related self is hypothesized to evolve by a causal progression from employment across employment time via emotional and cognitive components. It is general in its formulation, meaning that it is independent of different types of work-related identities. This suggests that a work-related self is a higher-order construct (Law et al., 1998; Stajkovic, 2006) capturing basic psychological processes grounding the associations between occupational work and the self; the self, which *always* strives for a meaning. This phenomenon is conceptualized as a knowledge structure resulting in a *personal*, autobiographical work-related experience of “*my* work” as opposed to the general concept (declarative knowledge) of “work” (Kihlstrom and Klein, 1994; Wilson and Ross, 2003; Conway and Holmes, 2004; Knez, 2014). Accordingly, this model relates to the Ashforth et al. (2008, p. 327) questions of “What is it?” and “How does it evolve?” even though they did “not consider personal identities.”

## Identity and Self

Identity directs a person “to behave in certain ways” (Vignoles et al., 2008, p. 1166) and comprises two basic categories of personal and social identity. The former refers to our individual body and mental experiences. I am, in other words, a separate physical and psychological entity compared to other people, beings and things; an understanding that is constant over time. The latter type of identity refers to our membership of, belonging to, different social groups and contexts. Thus the social identity is associated with “group membership, group processes and intergroup behavior,” and personal identity is associated with “close personal relationships and idiosyncratic attributes” (Hogg, 2003, p. 463). The type of identity that is of primary interest in the present article is personal and collective *knowledge* (Kihlstrom and Klein, 1994) of being a member of an occupational work context; especially, personal experience as referring to the general model of work-related self. This is in agreement with “the self as a knowledge representation” perspective, meaning that self-related information is apportioned across declarative memory (Klein and Loftus, 1993; Knez, 2014).

Psychological research in this area is extensive, including findings on identity in relation to its: (1) Form, content and organization; (2) Regulation and control; (3) Evaluation, motivation and emotion; (4) Interpersonal aspects; and (5) Phylo- and ontogeny-development (Leary and Tangney, 2003; see also Swann and Bosson, 2010 for a review). Personal and social identities can also be said to include and accommodate private and collective self, respectively, comprising self-concept and self-image, and the sense of continuity of “who I/we are” over time (Brewer and Gardner, 1996). Social identity can furthermore be characterized as a relational self, referring to personal ties such as a partner-relationship (Sedikides and Brewer, 2001); addressing the discussion of the primacy of the intra- or the interpersonal self (Tice and Baumeister, 2001).

Accordingly, we define, perceive and interpret ourselves by the operations of two basic psychological mechanisms of: (1) *Being* unique (*my* body, memories, thoughts, opinions, feelings, experiences, sensations); and (2) *Belonging* to a group/social relationship (I am, e.g., journalist, father, woman, student, Englishman, fisherman).

## Self and Memory

According to current developmental psychology, body-related self-consciousness starts at between 18 and 24 months of age (Anderson, 1984). Development of self-consciousness in the cognitive sense starts a little later and in parallel with language and episodic memory development (Fivush, 2011). Furthermore, this initiates the ability to travel in an inner mental world (McCormack and Hoerl, 1999). Consequently, young children up to 24 months of age are able to remember specific events and places, but these experiences will not be interpreted from the I/me perspective until the age of 3–4 years.

Human long-term memory comprises two declarative/explicit memory systems (Squire, 1992). The episodic memory system contains the self-related (personal) information in a time and space perspective and semantic memory system stores all fact-related (impersonal) information (Tulving, 1972). Knowing that Stockholm is the capital of Sweden and that two plus two is four is hence stored in, and retrieved from, my semantic memory system, while my summer vocation memories from Stockholm might be recollected from my episodic memory system, or for that matter the information about when and where I learned that two plus two is four.

Different types of consciousness are also related to these memory systems (Tulving, 1985; but see also Henke, 2010). That is, I *know* and by that I am aware of (knowledge-related, *noetic*, awareness) that Stockholm is the capital of Sweden. I am also self-conscious (*autonoetic* awareness) about my summer experiences in Stockholm; in other words, I *remember* these personal experiences. Accordingly, when we talk about personal involvement we retrieve that information from episodic memory (e.g., “I *remember* when I defended my Ph.D. thesis.”), while impersonal facts are recollected from semantic memory (e.g., “I *know* what a Ph.D. degree means.”).

The relationship between memory and the self has long engaged various philosophical and psychological schools (see Klein, 2012 for a review). One could, in general terms, say that the relationship between memory and the self is reciprocal. That is, we are what we remember, and vice versa. Self is, therefore, a product of its past, its memories (Locke, 1690/1849; Grice, 1941; Klein, 2001). Further, remembering presupposes a self, an agent that remembers/knows (James, 1890/1950; Wheeler et al., 1997; Suddendorf and Corballis, 2007); as pointed out by Tulving (1985, p 15): “It is the self that engages in the mental activity that is referred to as mental time travel: there can be no travel without a traveler.”

Autobiographical memory handles relationships between memory and the self. It is a system, a knowledge representation (Kihlstrom and Klein, 1994) that manages the psychological blocks of the self, such as coherence in the self across time, its social information base, and self-targeted behaviors and problem solving (Bluck et al., 2005; Conway, 2005). The autobiographical information is distributed across declarative memory as impersonal information (Ph.D. degree as a concept is stored in semantic memory) and personal information (when and how I defended my Ph.D. thesis is stored in episodic memory). More precisely, within episodic memory system the self-related memories are associated with the sense of personal identity across time and space, and within semantic memory system the self-related memories are associated with the factual and trait- based self-knowledge (Klein and Gangi, 2010; Klein and Lax, 2010). In the words of Klein (2013, p. 3): “We are acquainted with semantic pastness indirectly via inference, whereas our acquaintance with episodic pastness is directly given as the feeling that we are re-living our past.”

The experiential, phenomenological dimension of these “re-living” operations is often characterized as a personal life story (Habermas and Bluck, 2000; Fivush, 2008). Thus, when we remember we tell a story, in a broad sense. It contains different types of themes/chapters (e.g., work, education, relationships, and places) around which the personal experience information is grouped and retrieved (Williams et al., 2008; Knez, 2014). For example, if I recall “my first girlfriend” I can recount where and how we met, her name, what she looked like, where she lived, what music we used to listen to, what friends we hung out with, etc (see Prebble et al., 2013 for a recent account of self and memory).

The self as “a knowledge representation” perspective does also imply that we may have several “context-specific” selves (McConnell, 2011) and that “autobiographical self is not a mere list of episodic memories, but more likely a narrative structure that includes the temporal and causal relations among remembered events” (Kihlstrom, 2012, p. 367). In the words of Gao and Riley (2009, p. 325): “…knowledge has a place in the individual’s cognitive identity structure and, consequently, a role in identity formation.” Finally, the neural structure accounting for the self-referential information processing is the medial prefrontal cortex (D’Argembeau and Salmon, 2012).

## Work-Related Identity

Given the above, we could say that humans unlike animals (Markowitsch and Staniloiu, 2011) have two basic psychosocial needs of: (1) *Distinguishing* us from others in order to preserve the personal self, the personal story and its memories (Brewer, 1991); and (b) *Belonging* to a social group, a social context, in order to be part of the collective self, the collective story and its memories (Brewer and Gardner, 1996). Further, and according to social identification theory (Tajfel and Turner, 1979, 1986; Pratt, 1998; Jackson, 2002; van Dick, 2004; Ellemers et al., 2004) the following three mechanisms operate on behalf of a healthy self and social identity: (1) Thriving toward positive self-evaluation; (2) Linking social affiliations to self-concept and social identity; and (3) Retaining and strengthening positive social group identity, by in- vs. out-group comparisons. These additionally interact (Boros et al., 2011) across: (1) *Knowledge* related to person’s social identification; (2) *Emotional* links developed to various social groups; (3) *Valuations* of in- vs. out-group distinctions; and (4) *Behavioral* sets tied to different social identifications.

Organized labor is, as mentioned above, essential for a person’s self-worth, meaningful and healthy life (Ashforth and Mael, 1989; Fine, 1996; Gini, 1998; Paul and Moser, 2009). For that reason it constitutes a pivotal social context within which individuals seek multidimensional (Ashforth and Mael, 1989) loyalties (Reichers, 1985; Becker et al., 1996); coalitions labeled as organizational, workgroup and professional identity (Causer and Jones, 1996; Cappelli, 2000; Day et al., 2006; Johnson et al., 2006; Pate et al., 2009). Accordingly, the multiple self-concept representations (Neisser, 1988; McConnell, 2011) of professional identity are related to personal identity (“My profession, career, as a lecturer.” = personal self) and working group (“We lecturers working at the Department of Psychology.”) and/or organizational identities (“We lecturers working at the Faculty of Behavioral Sciences.”) are related to social identity (collective self).

Thus and in an organized labor context, individual identity is associated with self-related interests and motivation (personal self). It generates behaviors that mirror personal attitudes, aspirations, and goals (Ybarra and Trafimow, 1998). Social identity, on the other hand, is associated with attitudes, norms and behaviors related to a group’s identity, to its collective self (Hogg, 2006; Jackson et al., 2006). Accordingly, this is in line with the self-categorization theory (e.g., Turner et al., 1994) suggesting a distinction between personal identity (personal self) and social identity (collective self) indicating that individual and social identity incorporates two functionally independent cognitive structures of self-representations (personal vs. collective), leading to different self-related interpretations and definitions (Sedikides and Brewer, 2001). This does *not*, however, suggest that the private and collective selves are unconnected, but that: “…one system can operate independently of the other, though not necessarily as efficiently as it could with the support of the other intact system.” (Tulving, 1983, p. 66); meaning that “self-concepts can take different forms at different levels of abstraction” (Onorato and Turner, 2004, p. 276)

We also strive for positive work-related allegiances (Gecas, 1982; Turner, 1982; Bartel and Dutton, 2001), as positive identity construction may, for example, enhance well-being (Caza and Wilson, 2009) and creativity (Cheng et al., 2008), as well as promote career adaptation/development (Ibarra, 1999). According to Dutton et al. (2010) four theoretical perspectives capture the positivity in work-related identity; namely, virtue perspective (e.g., courage, humanity, justice), evaluative perspective (feelings of self-regard increasing/maintaining the self-worth), development perspective (progress/adaptation in identity development), and structural perspective (balance in multidimensional loyalties).

Yet, one important factor to consider is that organizational and workgroup identities are not everlastingly settled. Persons can sometimes feel greater affinity with their workgroup compared to the organization and sometimes vice versa, depending on the strength of the identification and positioning of *we* and *they* classifications that evolve and change across time and workplace (Wagner and Ward, 1993; van Dick, 2004).

Finally, work-related identification implies not only individual and social classifications, but relationship patterns too. These are collections of knowledge structures (scripts/schemas stored in semantic memory) related to particular situations; such as, “attending a business meeting.” A script/schema of this kind gives us all the different social processes that usually occur when we are attending a business meeting. This mental representation serves as a guide for our perception, interpretation, and understanding of the situation at hand and the people involved (Bartlett, 1932; Higgins and Bargh, 1987). When it comes to work-related identity, a script/schema would then *instruct* our perceptions and interpretations of in- and/or out-group interactions and situations (Baldwin, 1992; Terry and Hogg, 1996).

## Work-Related Identity in Practice

Given the above, we may predict that similar values, beliefs and standards will be found in individuals sharing a strong in-group identity (Tajfel, 1978; Mael and Ashforth, 1992). Previous research has indeed indicated that the stronger the group identification an individual feels, the more that group will influence his/her attitudes and behaviors (Hogg and Abrams, 1988). Employees with weak organizational identity will, for example, not engage in the instructions and information that the organization communicates, because they have too weak organizational identity (Mobley et al., 1979). They simply do not share organizational values (Johnson and Jackson, 2009). It has also been shown that social group identification is central to Internet-based work, although individuals never meet in reality (Michinov et al., 2004). Work-related identity has also been indicated to mediate effects of organizational justice on trust and cooperative behavior (Johnson and Lord, 2010).

Accordingly, it is difficult to fully understand employees’ work-related behaviors if we do not take into account their occupational identities (Vondracek et al., 1986; van Dick, 2004; Edwards, 2005; Wayne et al., 2006; Gao and Riley, 2009; Greenhaus et al., 2012; Ravasi and Canato, 2013; Brown, 2014). Why? This is because personal and social identifications are profoundly involved in formations of work-related self-image, self-esteem, motivation, task perception and valuation of future professional perspectives (Kelchtermans, 1993), as well as with operational efficiencies and strategic agreements (Sinclar, 2008). Concerning self-image and self-esteem issues, Lipponen et al. (2005) have indicated an impact of perceived prestige on work-group identification, showing that high group reputation is associated with strong group identification. Obschonka et al. (2012) have additionally suggested that entrepreneurship may be a function of: (1) Social norms in individuals with a strong work-group identity; or (2) Personal initiative and control in individuals with weak work-group identification.

### Adult Development and Work-Related Identity

Formation of work-related identity coincides with adult identity establishment (Marcia, 1980; Blustein et al., 1989), stabilized around 20–30 years of age (Arnett, 2000, 2004; Plug et al., 2003). It consists, roughly, of four phases (Savickas, 1985): (1) Diffusion (“I don’t really know what I want to do/*be*.”); (2) Precursor (“Well I’ll check this type of work.”); (3) Moratorium (“I’ll try to develop within this profession.”); and (4) Performance/commitment (“I know what I want to do/*be”*). The formation of a work-related identity is assumed to be “completed” during the person’s 30 s (Plug et al., 2003; Arnett, 2004).

This process is not just about a young person entering the world of employment but is also related to voluntary (personal motivation, aspiration, and ambition) and involuntary (institutional and situational constraints) work-related transitions (Foud, 2007). Luyckx et al. (2010) have shown that strong personal identity in young adults correlates highly with work-related commitment and low risk of work-related depression. This suggests that the formation of a personal identity goes hand in hand with psychosocial adjustments (Kroger, 2007), and that work-related identity is an important part of the formation of an adult identity (Skorikov and Vondracek, 2007).

### Demographic Variables and Work-Related Identity

Men put more emphasis on salary, power, and career. They also spend more time working (Greenhaus et al., 2012); which female and male managers also do (Brett and Stroh, 2003). Gender is also involved in work-related identity issues (Sluss and Ashforth, 2007). According to social identity theory, we might predict that gender as a homogeneous group (in-group) would show strong group identification; e.g., operationalized as group commitment and cohesion (Williams and O’Reilly, 1998; Swann et al., 2004). Van Knippenberg et al. (2007) have shown, however, that gender group identification may be more related to beliefs about gender related diversity/homogeneity than to group homogeneity *per se*. They reported that gender diversity (male + female group composition) compared to gender homogeneity (male vs. female group composition) was associated with higher levels of work-group identity.

Becoming a part of a new social context and hence acquiring new work-related identities has also been investigated in studies addressing globalization issues, indicating different career paths across geographical and cultural boundaries (Cohen et al., 2011; Flum and Cinamon, 2011) that may be linked with age and ethnic changes in society. This has, for example, already happened in Great Britain (Kenny and Briner, 2007). It is estimated that 50% of the future workforce will be composed of minority ethnic groups (which is only 8% of the population) due to these groups’ younger age composition (Cabinet Office Strategy Unit, 2003).

### Work-Related Satisfaction and Work-Related Identity

Previous findings have shown that organized labor is related to physical and mental health and, conversely, that unemployment is associated with physical and mental illness (Murphy and Athanasou, 1999; Friedland and Price, 2003; Paul and Moser, 2009). It has also been indicated that personal well-being and happiness take some time to recover after a period of unemployment (Lucas et al., 2004). Correspondingly, work-related identity has been reported to associate with work-related support (Mael and Ashforth, 1995), commitment (Baruch and Cohen, 2007), performance and motivation (Haslam et al., 2000), group relations (van Knippenberg, 2003), organizational mergers (Haslam, 2001), changes (du Gay, 1996), learning (Collin, 2008), organizational justice (Olkkonen and Lipponen, 2006), and with the appreciation of the work *per se* (Collin et al., 2008). Escartín et al. (2013, p. 182) have also indicated that “the more employees identified with their group, the less likely they were victims of bullying.” Thus, work-related satisfaction, in general terms, is strongly linked to work-related identity (Fenwick, 2004; Kirpal, 2004).

Strong work-related identity can, however, be difficult to change in cases of reorganization, for example, because it takes some time to break up and create new identities and loyalties (van Dick et al., 2006; van Vuuren et al., 2010). A process that can also generate stress among employees (Jetten et al., 2002) and lead to decreased confidence, motivation, commitment, and performance (Haslam, 2001). Employees may, in other words, lose their personal feelings of control, meaningfulness and identification with the workplace (Ashforth, 2001).

Concerning the relation between work-group identity and work-group size, some findings have indicated a tendency to a stronger identification with smaller rather than larger work-groups (van Knippenberg and van Schie, 2000; Riketta and Dick, 2005). In particular it is shown that identification with a group correlates highly with work-related job satisfaction (van Knippenberg and van Schie, 2000). This is in line with some research on work-group size that has shown that the smaller the group, the stronger the workgroup identification (Mullen, 1991; Lipponen et al., 2005).

Work-related identity has also been found to mediate individual work-related learning with adaptation (Sfard and Prusak, 2005; Kirpal et al., 2007). Employees’ work-related self-assessment has been shown to increase as a result of leaders’ work-related self-sacrifice coupled with strong work-related identity in employees (Cremer et al., 2006). Work-related engagement (Buchanan, 1974; Staw and Salancik, 1977; Mowday et al., 1982), motivation and performance have also been reported as being associated with work-related identity (van Knippenberg, 2000; Mathews and Shepherd, 2002).

Motivation is especially important in this context. It has been reported that people with a weak group identity “mimic” others’ behaviors, while individuals with a strong group identity contribute more to the group if others “slip by” (Fishbach et al., 2011). This implies that when a group weakens its position, individuals with a weak group-identity will withdraw from the group so as not to risk something personal, while individuals with a strong group-identity will sacrifice more in order to maintain the status of the group (Tajfel and Turner, 1979).

Previous research has furthermore indicated an association between work-related identity and health/well-being (Haslam et al., 2009; Wegge et al., 2012). Strong occupational identity can also function as a buffer against stress (Haslam, 2001). Wegge et al. (2006) reported a relationship between work-related depression, satisfaction and hectic work environment, showing an association between strong work-related identity and a small number of cases of depression and high occupational satisfaction. A link between work-related identity and burnout has also been indicated (Luyckx et al., 2010), suggesting that burnout may increase when professional identity decrease (Edwards and Dirette, 2010).

Two other important factors related to work-related identity are perceived justice and economic earnings. These have been shown to have a strong effect on employee behavior and engagement, especially with regard to extra work that an employee is willing to perform (Blader and Tyler, 2005, 2009). Thus, it is vital for an employer to behave fairly toward employees and financially reward their work. In line with this, Cremer (2006) has shown that individuals with a strong organizational identity will be disappointed when treated unfairly, leading to acts of revenge against management when perceived disappointment is “sufficiently” great. Other results have also indicated that negative emotions may be associated with unfair treatment (Tripp and Bies, 1997), sabotage (Ambrose et al., 2002), strikes (Leung et al., 1993), and revenge (Skarlicki and Folger, 1997).

It may thus be the case that employment as a social context is an important factor for individuals’ work-related meaning and satisfaction (Aamondt, 2009), and that it is strongly related to the phenomenon of work-related identity (Smith et al., 1969; Hackman and Oldham, 1980). Satisfied employees are usually more often at work than those who are not (Johns, 1997; but see Diestel et al., 2013), perceiving their occupational work as a part of themselves; as *my castle*, an analogy with the expression of “my home is my castle” (van Dick, 2001). Accordingly, people with a strong work-related identity will perceive their occupational work in terms of *my work* (*my castle*); as Gini (1998, p. 714) pointed out: “Descartes was wrong. It isn’t *Cogito ergo sum*, but, rather, *Laboro ergo sum*. We need work, and as adults we find identity and are identified by the work we do.”

## Recommendations for Future Research

Given that we strive for both personal and social identities (Sedikides and Brewer, 2001), one important issue that previous research has as yet not fully addressed is whether employees can have multiple, equally strong occupational identities (Pate et al., 2009); and how these types of identity are linked to personal and social selves (Onorato and Turner, 2001; Sedikides and Gaertner, 2001; Smith et al., 2001; Spears, 2001; Tice and Baumeister, 2001; Ashforth et al., 2011) and personal careers (Di Fabio and Maree, 2012; Di Fabio and Saklofske, 2014; Di Fabio and Kenny, 2015)? One way to approach this question within work and organizational research (Blader et al., 2007) would be to study associations between personal, relational and collective selves with types of work-related identity (Reid and Deaux, 1996; Farmer and Van Dyne, 2010), and how these constructs are organized in autobiographical memory (Knez, 2006; Williams et al., 2008).

Cognitive, emotional and behavioral components of personal and social identification (Kidd, 1998; Ellemers et al., 2004; Boros et al., 2011) should also be included in future analyses, especially related to the processes of construction and maintenance of an identity. Furthermore it is important to focus on work-related identity and satisfaction in relation to work-related justice (Johnson and Lord, 2010), leader/employee perspectives (George, 1995; McLain, 1995; Siegall and McDonald, 1995) and demographic variables (Kim and Gelfand, 2003; Kavanaugh et al., 2006; Kenny and Briner, 2007; Sluss and Ashforth, 2007; Cohen et al., 2011).

Finally, some of the previous research on identity and memory (Knez, 2005, 2006, 2014) has recognized concepts of time and attachment/belonging/closeness as two central factors in identity formation. It would therefore be of interest to transfer these theoretical ideas and empirical results to the context of work-related identity. Given this, I will below sketch a general model of the phenomenon of work-related self.

## Toward a Model of a Work-Related Self

“From the approximate ages of 21–70 we will spend our lives working… like Sisyphus we are all condemned to push and chase that thing we call our job, our career, our work all of our days” (Gini, 1998, p. 707). Work/employment is “a pervasive life domain and a salient source of meaning and self-definition” (Dutton et al., 2010, p. 265). Accordingly, the phenomenon of occupational work is central to human life, involving considerations of purpose and meaning(fullness) of work (Hackman and Oldham, 1976; Schwartz, 1999; Wrzesniewski et al., 2003; Heine et al., 2006; Grant, 2008; Rosso et al., 2010); that may affect, for example, work-related motivation (Roberson, 1990), well-being (Ryan and Deci, 2001), engagement (May et al., 2004), and career development (Dik and Duffy, 2009). Hence: “The more involved one is with the job, the more difficult is to dissociate oneself… from that job” (Rosso et al., 2010, p. 97).

Given that: (1) The self is a “complex system of active and interactive self-organizing processes” (Mahoney, 2002, p. 747); (2) Autobiographical memory manages the psychological blocks of the self, involving several context-specific selves and integrating emotional and cognitive processes in a goal-directed manner; (3) Individual and social identity incorporates two functionally independent, but interacting, cognitive structures of self-representations; (4) Individual self is the “home base” for self-definitions constituting the “essence of the person,” because “the level of individualism is relatively stable and invariant,” meaning that it is “less amenable to variation due to culture” (Sedikides and Gaertner, 2001, p. 19,20), I suggest a *higher-order* work-related identity construct, the *working-self*, capturing basic psychological, individual mechanisms accounting for the occupational work-self associations. This is in accordance with the definition of the “healthy and stable self” (Conway et al., 2004; Klein et al., 2004); a perspective that has, as far as I know, not been previously addressed by any account in occupational and organizational psychology regarding the phenomenon of work-related identity.

### Model Components

Previous findings in identity, self and memory research have shown that the course of self-formation involves factors of *time, attachment/belonging/closeness, coherence, correspondence, temporality* (mental time), *reflection* and *agency* (e.g., James, 1890/1950; Klein et al., 2004; Conway, 2005; Knez, 2014). These, in addition, interplay with social contexts of, for example, geographical location (Knez, 2014) and employment; a “work theme” (Williams et al., 2008). Given that affect plays a crucial role in meaning of life and identity formation (King et al., 2006; Burke and Stets, 2009) and that previous research on work-related identity has predominantly been focused on cognitive aspects (see Edwards, 2005 for a discussion), the present model will include (as suggested by, for example, van Dick, 2004; Edwards, 2005; Burke and Stets, 2009; Rosso et al., 2010) both “employee’s thoughts and feelings that have reference to himself [herself – added]” (Rosenberg, 1979, p. 7).

**FIGURE 1:**
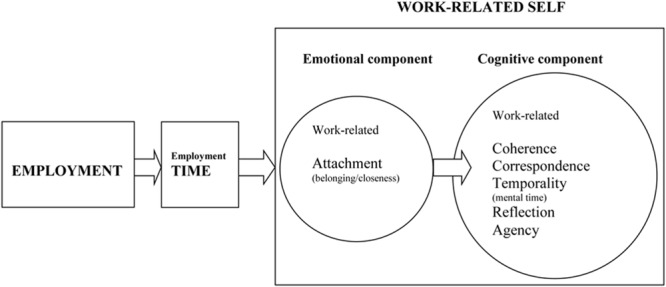
**Causal formation of work-related self; from employment across employment time to work-related emotional (attachment/belonging/closeness) and cognitive (coherence, correspondence, temporality/mental time, reflection, agency) components**.

The conceptual model of work-related self (see **Figure 1**) encompasses *time* and two basic *psychological* elements: (1) Emotional component comprising the process of work-related attachment/belonging/closeness; (2) Cognitive component comprising the processes of work-related coherence, correspondence, temporality/mental time, reflection, and agency.

Time is included due to its relation to work orientation (Rosso et al., 2010) and because identity “…depends, in fundamental way, on our capacity to represent the self as a psychological coherent entity persisting through time” (Klein et al., 2002, p. 353). Similarly, Kant (1787/1929) defined time as one of the basic formats for the structuring of human experience (see also Klein, 2014a for a discussion about the phenomenon of “sameness,” that is, how we across time feel that we are the same?). In addition, Goodman et al. (2001, p. 507) pointed out that “there is surprisingly little research on time” in organizational life research.

The model is general in its formulation, meaning that it is *independent* of different types of work-related identities. Accordingly, work-related self is a *higher-order* construct (Law et al., 1998; Stajkovic, 2006) that captures the basic psychological processes grounding the associations between the occupational work and the self. This phenomenon is conceptualized as a knowledge structure comprising the *personal* information (Kihlstrom and Klein, 1994; Wilson and Ross, 2003; Conway and Holmes, 2004; Knez, 2014) of “*my* work” as opposed to the declarative knowledge of the “work” concept. In the words of Kihlstrom et al. (2003, p. 69): “The self is a mental representation of oneself, including all that one knows about oneself. The *I* who knows the *me* is the same *I* who knows everything else, and the mental representation of this knowledge is no different, except perhaps in intimacy and richness, than is the mental representation of anything else I know.”

The main function of the work-related self is to emit the autobiographical experience of “*my* work” (*my castle*; van Dick, 2004). As can be seen in **Figure 1**, work-related self is grounded in the social context of *employment* across *time*; it is thus “an individual work identity” (Walsh and Gordon, 2008) based on the interactions between work and work-related identifications (Kira and Balkin, 2014). Its main function is to guide personal reminiscence and self-knowing consciousness related to the work context; that is, to the autobiographical theme of occupational work (Conway, 2005; Williams et al., 2008) distributed across declarative memory as impersonal (employment/work as a concept) and personal (my employment/work) information (Klein et al., 2004; Bluck et al., 2005) about our work experiences. The two psychological forces accounting for the construction of the work-related self are the emotional and cognitive components involving the self-formation processes of *attachment/belonging/closeness, coherence, correspondence, temporality* (mental time), *reflection* and *agency* (e.g., James, 1890/1950; Conway et al., 2004; Klein et al., 2004; Conway, 2005; Knez, 2014).

The processes defining emotional and cognitive components, below, relate to the fundamental question of “who one is?” paralleling the concepts of sensebreaking/sensegiving and/or claiming/granting in organizational identity construction literature (Weick, 1995; Pratt, 2000, 2001; Wrzesniewski et al., 2003; Ashforth et al., 2008).

#### Emotional Component

The impact of a caregiver-infant relationship on infancy and early childhood has been addressed by attachment theory (Sedikides and Skowronski, 2003). This frame of reference cannot, however, be converted analogously from the caregiver-infant relationship to the vocational behavior context; because, the latter indicates a *one-way closeness* between a person and its occupational work as opposed to the former which designates a reciprocal link between an infant and a caregiver (Bowlby, 1988). Attachments, in general terms, are: “relationships…to particular people whom we love … and sometimes to particular places that we invest with some loving qualities.” (Marris, 1982, p. 185); meaning that “individuals identify by seeking to experience a sense of pride, warmth, or affirmation” (Ashforth et al., 2008, p. 343; see also Harquail, 1998). Thus, if we assume that “place,” in Marris’ (1982) words, correspondence to “employment,” we could subsequently posit a *one-way closeness* (attachment) between a person and its employment (see Knez, 2014 for this type of argument), indicating an employee’s affection to its occupational work (Meyer and Allen, 1991; Johnson and Jackson, 2009).

#### Cognitive Component

The concept of *coherence* signifies “rationality” (Hammond, 2007) or “conservatism” (Greenwald, 1980) of the self (continuity in the self across time, in James’ terms), and *correspondence* refers to the on-going processes of “accurate/adaptive” interactions between the self and its contexts (Conway et al., 2004). Furthermore: “We feel and act about certain things that are ours very much as we feel and act about ourselves.” (James, 1890, p. 291). These words capture the two “necessary components” of the healthy self and its processes of autobiographical recollection as suggested by Klein et al. (2004); the third one is self-temporality (*temporality/mental time*). Thus, and in order to coherently and correspondingly ground the working-self across employment time, the person must also be aware of, and reflect (*reflection*) upon, her/his (*agency*) mental states as related to the work contexts; standpoints that also coincide with Erikson’s (1968) reasoning about the defining-and-meaningful-identity (the self) comprising components of inner continuity, consciousness and agency across time.

In sum, a general framework for the phenomenon of work-related self is sketched. This account proposes (see **Figure 1**) that the transformation of “work” (declarative fact with accompanying noetic consciousness) to “work of mine” (personal fact with accompanying autonoetic consciousness) is due to an amalgamation of employment, employment time and psychological processes of work-related attachment/belonging/closeness, coherence, correspondence, temporality, reflection and agency. Accordingly, the model predicts that the organization will “in effect, been incorporated into the self-concept… the organization becomes a part of the individual’s self-concept.” (Edwards, 2005, p. 213). Practically, this means that when a manager, for example, plans to re-construct a department or teamwork the employees’ work-related selves (identifications) will matter (van Dick, 2001).

Given that managers “are people who do things right” (Bennis and Nanus, 1985, p. 221) related to functions of planning, budgeting, organizing, staffing, controlling, and problem solving (Kotter, 1990) and that role modeling plays a part in identity construction (Shapiro et al., 1978), we may say that *I*, for example being a senior female manager and a role model (Sealy and Singh, 2009), across employment *time* have evolved an *attachment* (belonging/closeness) toward my position (*employment*) and by that have initiated my *work-related self*. *Reflecting* upon these experiences (memories) of mine (*agency*) *I* remember (I’m *self-knowingly* consciousness about this personal chronicle) the date (inner *temporality*) when *I* became a manager (*coherence*) and by that a role model for other women, struggling with “rigid and male-dominated hierarchies” (Sealy and Singh, 2009, p. 284).

As a consequence of all this, *I* will plan to increase the “pool of available female role models at board director level” (Sealy and Singh, 2009, p. 285) [an accurate *correspondence* with the on-going present; indicating “congruence between self-at-work and one’s broader self-concept” (Rousseau, 1998)]. So, when *I* think/speak about my work *I* mentally re-live the experiences of “my castle” (van Dick, 2004); meaning that *I* feel good about my work, as well as that my work is a part of myself because autobiographical memory by definition “entails a mental representation of the self” (Klein, 2014b, p. 19).

## Conclusion

Accordingly, the model hypothesizes (see Knez, 2005, 2014), that across employment time we evolve an emotional tie to our work (prediction: the more time the stronger emotional link). Secondly, given that the emotion may modulate better retention in episodic memory (Canli et al., 2000), the model also predicts that the emotional connection may precede the cognitive one because personal memory (in this context, “work of mine”) may be more easily recalled due to its emotional connotations stored in the autobiographical knowledge base (Welzer and Markowitsch, 2005).

In line with Knez (2014, p. 170) reasoning, we could conclude that how *I* (the knower; the ontological self) ground *my* working-self (the known; the epistemological context-specific self) requires that: (1) I across employment time evolve an *emotional tie* (attachment/belonging/closeness) to my work; (2) I represent my working-self across employment time as a *coherent* self, (3) appropriately *corresponding* to the immediate ongoing work-related reality, and (4) that I am *reflective* upon and (5) aware of me as *possessing* my inner work-related world in a (6) *time-related* unfolding of my work-related experiences. All this is done within the context of a goal-driven autobiographical representation of the working-self.

Finally, this is no more than a conceptual model, which means that several studies are needed to test these ideas in more empirical detail.

## Author Contributions

The author confirms being the sole contributor of this work and approved it for publication.

## Conflict of Interest Statement

The author declares that the research was conducted in the absence of any commercial or financial relationships that could be construed as a potential conflict of interest.
